# Over-the-counter Medication Use during Pandemic: Lessons Learned from Covid-19 in Lithuania

**DOI:** 10.1093/eurpub/ckac131.083

**Published:** 2022-10-25

**Authors:** I Bikniūtė, M Paric, T Clemens, M Commers, H Brand, J Vladičkienėa, RJJ Elands

**Affiliations:** 1 Department of International Health, Maastricht University, Maastricht, Netherlands; 2 Department of Social Medicine, Kaunas University, Kaunas, Lithuania; 3 Historical Demography and Family History, Radboud University, Nijmegen, Netherlands

## Abstract

**Background:**

Despite the growing awareness about nonprescription misuse of anxiolytics, there is not much evidence about people’s behavior during the covid-19 lockdown when access to healthcare specialists was limited. Over the counter drug use has risen sharply in the past decades among college students and junior health care workers, yet, there are few studies reporting on the use of nonprescription medicine groups during the COVID-19 pandemic.

**Methods:**

A cross-sectional study among 163 second-year medical students of the Lithuanian University of Health Sciences was conducted in Kaunas, Lithuania with self-reported measures of anxiety and insomnia and comparing nonprescription medicine use for anxiety and insomnia before and during the covid-19 pandemic.

**Results:**

A near two-fold increase in the prevalence of anxiety and insomnia among Lithuanian medical students was reported during the covid-19 pandemic compared to before the onset of the pandemic (p < 0,001). The use of nonprescription medication increased during the pandemic (p < 0,001), in particular anxiolytics (p < 0,05). Once-weekly anxiolytic medication use increased from 8,0% before the pandemic to 14,7% during the pandemic. Regular nonprescription medicine use (2-3 times weekly) had more than tripled, from 2,4% to 9,2%. During the pandemic, almost a fifth of the respondents were increasingly searching for information on anxiolytic nonprescription medication online during the pandemic compared to before the pandemic.

**Conclusions:**

During the covid-19 pandemic, the prevalence of anxiety and insomnia increased among Lithuanian medical students, along with the practice of anxiolytic nonprescription medications. Lithuanian medical students increasingly practiced self-medication during the pandemic and found the information on nonprescription medication increasingly online, which offers opportunities for telemedicine.

**Key messages:**

• During the covid-19 pandemic, the prevalence of anxiety and insomnia increased among Lithuanian medical students, along with the practice of anxiolytic nonprescription medications.

• During covid-19, Lithuanian medical students increasingly retrieved information on nonprescription medication online instead of consulting a pharmacist, offering opportunities for telemedicine.

